# Geographic Access to Cancer Treatment in Japan: Results From a Combined Dataset of the Patient Survey and the Survey of Medical Institutions in 2011

**DOI:** 10.2188/jea.JE20170051

**Published:** 2018-11-05

**Authors:** Hirokazu Tanaka, Koichi B. Ishikawa, Kota Katanoda

**Affiliations:** 1 Department of Public Health, Graduate School of Medicine, The University of Tokyo, Tokyo, Japan; 2 Center for Public Health Sciences, National Cancer Center, Tokyo, Japan; 3 Center for Cancer Control and Information Services, National Cancer Center, Tokyo, Japan

**Keywords:** geographic information systems, health services accessibility, healthcare disparities, neoplasms, travel time

## Abstract

**Background:**

There has been no nationwide analysis of travel time for hospital admission in Japan. Factors associated with travel time are also unknown. This study aimed to describe the distribution of travel time for hospital admission of cancer patients and identify underlying factors.

**Methods:**

The individual data from the Patient Survey in 2011 were linked to those from the Survey of Medical Institutions in the same year, and GIS data were used to calculate driving travel time between the addresses of medical institutions and the population centers of municipalities where patients lived. Proportions of patients with travel time exceeding versus not exceeding 45 minutes were calculated. To analyze the data with consideration of both individual factors of patients and geographical characteristics of areas where patients lived, multilevel logistic model analysis was performed.

**Results:**

The analysis included 50,845 cancer inpatients. The majority of the cancer patients (approximately 80%) were admitted to hospitals located less than a 45-minute drive from their residences. The travel time tended to be longer for younger patients. The proportion of patients with travel time ≥45 minutes was lower among those with stomach or colorectal cancer (approximately 15%) than those with cervical cancer or leukemia (approximately 30%). The lack of designated cancer care hospitals in the secondary healthcare service areas was significantly associated with travel time.

**Conclusions:**

Selection of hospitals by cancer inpatients is affected by age, cancer sites, and availability of designated cancer care hospitals in the secondary healthcare service areas where patients live.

## INTRODUCTION

In Japan, because cancer has been ranked as the top cause of death since 1981, establishment of a cancer treatment system is an important public health issue.^1^ Since the Cancer Control Act was enacted in 2006, the Ministry of Health, Labour and Welfare has been promoting cancer prevention, efforts to reduce cancer deaths, equalization of cancer medical services, and early detection.^2^ To achieve these aims, 397 hospitals throughout Japan were designated as cancer care hospitals (hereinafter referred to as designated cancer care hospitals) in 2012 (398 hospitals in 2016). These facilities play an important role, mainly in the treatment of five major cancers common in the Japanese population (stomach, colorectal, liver, lung, and breast cancers) in each region.^3^ Meanwhile, 349 secondary healthcare service areas were also established in 2012. Because only 68% of them (236 areas) had designated cancer care hospitals, there might have been differences in the accessibility to cancer treatment among regions.^3^

The Japanese healthcare system is characterized by two major policies: universal healthcare coverage and free access.^4^ For this reason, the geographical factor is one of the primary obstacles to receiving cancer treatment. For cancer patients and their family members, commuting to a hospital distant from their residence would be a burden. However, the geographic distance that cancer patients, who generally have time to wait for treatment (unlike those with acute disease), must commute to medical institutions where they receive treatment is not well documented.^5^

The geographical distance from residences of patients to medical institutions has been assessed by calculating travel time (estimated time required to travel on public roads by car). Many studies have used travel time based on geographical information system (GIS) techniques to investigate access to health care.^6^^,^^7^ A systematic review showed that many of these studies reported an inverse correlation between travel time to medical facilities and various health outcomes (survival, mortality, quality of life, follow-up attendance, and usage of clinics).^6^ However, studies evaluating travel time were limited in Japan, especially for cancer treatment. For example, only one study reported that the mean travel time was 21.7 minutes for inpatients with breast cancer in Kyoto Prefecture,^8^ and we have identified no nationwide analysis of travel times in Japan. Although there is a report describing the distribution of travel time to a particular hospital in cancer patients,^9^ factors associated with travel time, such as the availability of designated cancer care hospitals in the secondary healthcare service areas and population density, the impacts of which may vary at the prefectural level depending on cancer sites, were not considered in that prior analysis.

In the present statistical analysis, travel time was estimated with data from national statistics to determine how far hospitals admitting cancer patients are located from their residences, while focusing on the characteristics of cancer patients and cancer sites. Furthermore, this study aimed to identify factors associated with travel time in consideration of differences among the prefectures.

## METHODS

### Data source

The individual data from the Patient Survey and the Survey of Medical Institutions in 2011 (both surveys are conducted once every 3 years) were obtained from the Ministry of Health, Labour and Welfare by applying for provision of individual survey data according to Article 33 of the Statistics Act.^10^^,^^11^ The obtained data contained no information that could be used for personal identification. The two datasets were linked to each other using facility codes that were included in these datasets, and we also added data on whether patients were admitted to designated cancer care hospitals and whether there were designated cancer care hospitals in the secondary healthcare service areas where the patients lived. The data included the sex, birth year, diagnosis, and address (municipality) of the patients. These data have been anonymized. Although the Patient Survey targets all patients in Japan, those in the Tohoku region affected by the Great East Japan Earthquake were excluded from the survey conducted in 2011. Patients with residences and who were treated at hospitals located in 12 secondary healthcare service areas consisting of only islands or isolated islands were excluded from the present analysis.

### Case classification

Diseases were classified according to the codes of the International Classification of Diseases, 10th revision. Cancers were divided into all cancers (C00–97, D00–09), stomach cancer (C16), colorectal cancer (C18–20), liver cancer (C22), lung cancer (C33–34), breast cancer (female, C50), cervical cancer (C53), and leukemia (C81–85, 88–96).

### Travel time

The travel time between the residences of the patients (municipalities) and hospitals was calculated using GIS data. In order to reflect geographic population distribution within a municipality, the travel time to each hospital was initially calculated according to 1-km mesh grid, and aggregated value at the municipality level was calculated by weighting each mesh with their population. The routes with the shortest travel time (without using toll roads, such as expressways) were selected, and driving speeds were set according to road types, such as 30 km per hour for local streets and 50 km per hour for wider national and prefectural roads. Six seconds were added to the travel time for crossing each intersection. The road network data used in this study were up-to-date as of April 2012. To calculate expected values of travel time in each municipality, we used the populations in the 1-km mesh areas based on the national population census in 2010. For cases in which travel time was estimated 90 minutes or over, we aggregated them as “90 minutes or over” because the estimation of travel time was uncertain.

### Statistical analysis

The outcomes were the number and proportion of patients with estimated travel time exceeding versus not exceeding 45 minutes according to age and sex. Age was divided into four categories: 0–39, 40–59, 60–74, and ≥75 years. Chi-square tests were used to examine travel time differences across sex. The availability of designated cancer care hospitals was determined according to whether they were located in the secondary healthcare service areas where the patients lived in 2012. Based on the national population census in 2010, the population density of each municipality was classified into five categories: top (0–20%), upper (21–40%), middle (41–60%), lower (61–80%), and bottom (81–100%).

We hypothesized that not only sex and age but regions (prefectures) where patients live may also effect travel time because prefectures have various feature in terms of geographic aspects and medical resources. To identify factors associated with longer travel time with consideration of prefectural level, multilevel logistic model analysis was performed with patient characteristics (ie, sex and age) as level 1 and characteristics of regions where patients lived (ie, the availability of designated cancer care hospitals and population density) as level 2, according to cancer sites. To assess variations in prefectural characteristics, median odds ratios (MORs) were calculated and compared according to cancer sites. STATA version 13/SE (Stata Corp, College Station, TX, USA) was used for data analysis and management.

## RESULTS

The analysis included 50,845 cancer inpatients. Table 1 shows the sex and age of the patients, as well as whether they were admitted to designated cancer hospitals. Of the patients included in the analysis (all cancers), 58.5% were male and 42.8% had been admitted to designated cancer care hospitals. Approximately 80% of the patients were aged over 60 years, and the proportions of those with stomach (87.9%), colorectal (85.8%), liver (91.4%), or lung (87.6%) cancers were high. On the other hand, the proportions of inpatients with breast (62.4%) or cervical (45.1%) cancer were relatively low, and those 40–59 years of age accounted for ≥30% of the inpatients with these cancer types. Regarding the distribution of all cancer patients according to the population density of their areas of residence, 16.8% of the patients lived in the top-category areas (0–20%), whereas 25.7% lived in the bottom-category areas (81–100%).

**Table 1.** Distributions of inpatients’ sex, age, and hospital

Variables	All cancer		Stomach		Colon/Rectum		Liver		Lung		Breast (female)		Cervix uteri		Leukemia	
	C00–D09		C16		C18–20		C22		C33–34		C50		C53		C81–85, 88–96	
Total	50,845		5,798		7,434		2,928		7,420		2,065		554		5,030	
Sex																
Male	29,759	(58.5)	3,852	(66.4)	4,241	(57.0)	1,966	(67.1)	5,098	(68.7)	—		—		2,678	(53.2)
Female	21,086	(41.5)	1,946	(33.6)	3,193	(43.0)	962	(32.9)	2,322	(31.3)	2,065		554		2,352	(46.8)
Age, years																
mean (SD)	69.7	(14.0)	72.6	(11.8)	71.9	(12.2)	72.9	(10.9)	70.9	(10.8)	63.6	(14.2)	57.1	(16.4)	63.9	(19.4)
≤39	1,597	(3.1)	69	(1.2)	77	(1.0)	28	(1.0)	50	(0.7)	84	(4.1)	91	(16.4)	543	(10.8)
40–59	7,216	(14.2)	629	(10.8)	980	(13.2)	224	(7.7)	875	(11.8)	694	(33.6)	213	(38.4)	864	(17.2)
60–74	21,587	(42.5)	2,338	(40.3)	3,100	(41.7)	1,200	(41.0)	3,596	(48.5)	794	(38.5)	154	(27.8)	2,000	(39.8)
≥75	20,445	(40.2)	2,762	(47.6)	3,277	(44.1)	1,476	(50.4)	2,899	(39.1)	493	(23.9)	96	(17.3)	1,623	(32.3)
Inpatient hospital																
DRCHs^a^	21,772	(42.8)	1,925	(33.2)	2,181	(29.3)	1,236	(42.2)	3,288	(44.3)	882	(42.7)	385	(69.5)	2,770	(55.1)
Others	29,073	(57.2)	3,873	(66.8)	5,253	(70.7)	1,692	(57.8)	4,132	(55.7)	1,183	(57.3)	169	(30.5)	2,260	(44.9)
SHSAs																
With DRCHs^a^	43,700	(85.9)	4,937	(85.2)	6,330	(85.1)	2,514	(85.9)	6,353	(85.6)	1,779	(86.2)	487	(87.9)	4,360	(86.7)
Others	7,145	(14.1)	861	(14.8)	1,104	(14.9)	414	(14.1)	1,067	(14.4)	286	(13.8)	67	(12.1)	670	(13.3)
Population density^b^																
Highest (top 0–20%)	8,528	(16.8)	884	(15.2)	1,286	(17.3)	492	(16.8)	1,280	(17.3)	324	(15.7)	90	(16.2)	848	(16.9)
Mid-high (top 21–40%)	8,916	(17.5)	986	(17.0)	1,236	(16.6)	492	(16.8)	1,315	(17.7)	395	(19.1)	120	(21.7)	909	(18.1)
Middle (top 41–60%)	9,533	(18.7)	1,010	(17.4)	1,355	(18.2)	582	(19.9)	1,430	(19.3)	406	(19.7)	116	(20.9)	1,008	(20.0)
Mid-low (top 61–80%)	10,810	(21.3)	1,307	(22.5)	1,645	(22.1)	661	(22.6)	1,464	(19.7)	415	(20.1)	114	(20.6)	1,021	(20.3)
Low (top 81–100%)	13,058	(25.7)	1,611	(27.8)	1,912	(25.7)	701	(23.9)	1,931	(26.0)	525	(25.4)	114	(20.6)	1,244	(24.7)

DRCHs, designated regional cancer hospitals; SD, standard deviation; SHSA, secondary healthcare service area.

^a^Designated by Minister of Health, Labour and Welfare in 2012.

^b^Population density of patients’ address municipalities calculated using census data in 2010.

The numbers in parentheses indicate percentages of categories within each variable.

The median travel time was 22.3 minutes (mean, 32.7 minutes; minimum, 4 minutes; and max, 90 minutes or over) among all cancer patients. Table 2 shows the results of travel time exceeding versus not exceeding 45 minutes according to sex and age. The travel time from the residences of the cancer patients to the admitting hospitals was ≥45 minutes in approximately 20% of all cancer patients. It tended to be longer in younger patients (the travel time was ≥45 minutes in 43% of men 0–39 years of age compared to 17% of men ≥75 years of age). For stomach and colorectal cancer, the proportions of patients with travel time of ≥45 minutes were low (approximately 15%). On the other hand, the proportions were somewhat higher in patients with cervical cancer or leukemia (approximately 30% each) than in those with other types of cancer. eTable 1 shows the percentages of patients whose estimated travel time exceeded 45 minutes across prefectures for all cancers. We observed variations of travel time across prefectures: the percentages of those with travel time exceeding 45 minutes ranged from 10% in Osaka Prefecture to 38% in Kochi Prefecture.

**Table 2.** Estimated cancer inpatients travel time by sex and age

Age, years	Sex	All cancer					Stomach					Colon/Rectum					Liver				
		C00–D09					C16					C18–20					C22				
		<45 mins		≥45 mins		χ^2^ test	<45 mins		≥45 mins		χ^2^ test	<45 mins		≥45 mins		χ^2^ test	<45 mins		≥45 mins		χ^2^ test
			%		%	*P* value		%		%	*P* value		%		%	*P* value		%		%	*P* value
All	Male	23,253	(78)	6,506	(22)	0.800	3,218	(84)	634	(16)	0.169	3,600	(85)	641	(15)	0.637	1,514	(77)	452	(23)	0.480
	Female	16,496	(78)	4,590	(22)		1,653	(85)	293	(15)		2,723	(85)	470	(15)		752	(78)	210	(22)	

0–39	Male	412	(57)	311	(43)	0.139	27	(77)	8	(23)	0.728	34	(77)	10	(23)	0.453	10	(56)	8	(44)	0.778
	Female	530	(61)	344	(39)		25	(74)	9	(26)		23	(70)	10	(30)		5	(50)	5	(50)	

40–59	Male	2,626	(71)	1,092	(29)	0.480	317	(75)	104	(25)	0.069	500	(81)	117	(19)	0.661	136	(74)	48	(26)	0.409
	Female	2,497	(71)	1,001	(29)		170	(82)	38	(18)		290	(80)	73	(20)		27	(74)	13	(33)	

60–74	Male	10,808	(77)	3,167	(23)	0.878	1,422	(82)	306	(18)	0.782	1,688	(84)	316	(16)	0.600	679	(76)	213	(24)	0.610
	Female	5,880	(77)	1,732	(23)		505	(83)	105	(17)		931	(85)	165	(15)		230	(75)	78	(25)	

≥75	Male	9,407	(83)	1,936	(17)	0.398	1,452	(87)	216	(13)	0.963	1,378	(87)	198	(13)	0.676	689	(79)	183	(21)	0.320
	Female	7,589	(83)	1,513	(17)		953	(87)	141	(13)		1,479	(87)	222	(13)		490	(81)	114	(19)	

Age, years	Sex	Lung					Breast (female)					Cervix uteri					Leukemia				
		C33–34					C50					C53					C81–85, 88–96				
		<45 mins		≥45 mins		χ^2^ test	<45 mins		≥45 mins		χ^2^ test	<45 mins		≥45 mins		χ^2^ test	<45 mins		≥45 mins		χ^2^ test
			%		%	*P* value		%		%	*P* value		%		%	*P* value		%		%	*P* value

All	Male	3,945	(77)	1,153	(23)	0.505	—		—		—	—		—		—	1,883	(70)	795	(30)	0.773
	Female	1,813	(78)	509	(22)		1,592	(77)	473	(23)		375	(68)	179	(32)		1,645	(70)	707	(30)	

0–39	Male	22	(81)	5	(19)	0.191	—		—		—	—		—		—	156	(54)	135	(46)	0.650
	Female	15	(65)	8	(35)		62	(74)	22	(26)		51	(56)	40	(44)		140	(56)	112	(44)	

40–59	Male	409	(69)	185	(31)	0.959	—		—		—	—		—		—	306	(63)	181	(37)	0.142
	Female	193	(69)	88	(31)		491	(71)	203	(29)		150	(70)	63	(30)		255	(68)	122	(32)	

60–74	Male	1,972	(76)	633	(24)	0.940	—		—		—	—		—		—	796	(72)	316	(28)	0.360
	Female	749	(76)	242	(24)		622	(78)	172	(22)		101	(66)	53	(34)		619	(70)	269	(30)	

≥75	Male	1,542	(82)	330	(18)	0.505	—		—		—	—		—		—	625	(79)	163	(21)	0.071
	Female	856	(83)	171	(17)		417	(85)	76	(15)		73	(76)	23	(24)		631	(76)	204	(24)	

Table 3 shows the results of multilevel logistic model analysis. The analysis with consideration of variations in prefectural characteristics did not identify sex as a statistically significant factor contributing to travel time exceeding 45 minutes for any cancer sites. The odds of travel time exceeding 45 minutes significantly increased with younger age; for instance, odds ratios for patients ≤39 years of age ranged from 2.32 (for lung; 95% confidence interval [CI], 1.16–4.65) to 4.48 (for leukemia, 95% CI, 3.54–5.66) compared to those ≥75 years of age. For all cancer sites, the lack of designated cancer care hospitals in the secondary healthcare service areas where patients lived was a statistically significant factor associated with travel time exceeding 45 minutes (odds ratio of stomach cancer, 2.10; 95% CI, 1.73–2.55). Regarding population density, the proportions of patients with travel time exceeding 45 minutes tended to be higher for those living in areas with a lower population density (*P* for trend <0.05 for all cancer sites). The highest MOR was 2.76 (for cervical cancer), whereas the lowest MOR was 1.55 (for colorectal cancer).

**Table 3.** Estimated odds ratios of cancer inpatients travel time whether ≥45 mins or not using multilevel logistic regression models

	All cancer			Stomach			Colon/Rectum			Liver		
	(*n* = 50,845)			(*n* = 5,798)			(*n* = 7,434)			(*n* = 2,928)		
	ORs	95% CI		ORs	95% CI		ORs	95% CI		ORs	95% CI	
Demographic level												
Sex												
Male	Reference			Reference			Reference			Reference		
Female	0.99	0.94	1.04	0.96	0.82	1.13	1.03	0.89	1.18	0.90	0.73	1.11
Age, years												
≤39	4.75	4.24	5.33	2.74	1.52	4.96	3.24	1.87	5.63	4.05	1.81	9.05
40–59	2.39	2.23	2.55	2.28	1.80	2.87	1.85	1.51	2.25	1.64	1.15	2.33
60–74	1.66	1.58	1.75	1.63	1.38	1.92	1.34	1.15	1.55	1.32	1.08	1.62
≥75	Reference			Reference			Reference			Reference		
(*P* for trend)	<0.001			<0.001			<0.001			<0.001		
Regional level nested by prefectures												
SHSAs												
With DRCHs^a^	Reference			Reference			Reference			Reference		
Others	2.02	1.90	2.16	2.10	1.73	2.55	1.77	1.49	2.11	2.77	2.15	3.57
Population density												
Highest (top 20%)	Reference			Reference			Reference			Reference		
Mid-high (60–80%)	1.39	1.27	1.54	1.64	1.17	2.29	0.94	0.70	1.26	1.42	0.94	2.15
Middle (40–60%)	1.58	1.41	1.76	1.25	0.85	1.83	0.91	0.65	1.26	1.20	0.76	1.90
Mid-low (20–40%)	3.18	2.83	3.56	2.34	1.62	3.39	1.78	1.30	2.45	2.07	1.31	3.27
Low (lower 20%)	9.29	8.25	10.45	5.54	3.80	8.07	4.74	3.41	6.58	6.67	4.17	10.7
(*P* for trend)	<0.001			<0.001			<0.001			<0.001		

Random effects												
Prefectures (*n* = 46)												
Standard deviation	0.241			0.269			0.208			0.253		
(Standard error)	0.053			0.079			0.061			0.086		
Median odds ratio	1.60			1.64			1.55			1.62		

	Lung			Breast (female)			Cervix uteri			Leukemia		
	(*n* = 7,420)			(*n* = 2,065)			(*n* = 554)			(*n* = 5,030)		
	ORs	95% CI		ORs	95% CI		ORs	95% CI		ORs	95% CI	

Demographic level												
Sex												
Male	Reference			—			—			Reference		
Female	1.02	0.90	1.16	—			—			1.04	0.91	1.19
Age, years												
≤39	2.32	1.16	4.65	2.55	1.42	4.59	3.18	1.51	6.68	4.48	3.54	5.66
40–59	2.65	2.19	3.21	2.77	2.01	3.80	1.45	0.76	2.77	2.50	2.03	3.08
60–74	1.91	1.67	2.19	1.62	1.18	2.23	2.28	1.16	4.49	1.81	1.52	2.15
≥75	Reference			Reference			Reference			Reference		
(*P* for trend)	<0.001			<0.001			0.039			<0.001		
Regional level nested by prefecture												
SHSAs												
With DRCHs^a^	Reference			Reference			Reference			Reference		
Others	2.03	1.72	2.39	2.55	1.87	3.47	2.75	1.41	5.34	2.84	2.32	3.49
Population density												
Highest (top 20%)	Reference			Reference			Reference			Reference		
Mid-high (60–80%)	1.40	1.08	1.82	1.30	0.82	2.06	1.19	0.52	2.72	1.41	1.06	1.88
Middle (40–60%)	1.82	1.36	2.44	1.14	0.68	1.91	2.52	0.97	6.58	1.88	1.37	2.58
Mid-low (20–40%)	3.83	2.83	5.19	1.93	1.13	3.31	5.48	2.00	15.0	3.58	2.56	5.00
Low (lower 20%)	12.6	9.17	17.2	5.84	3.39	10.1	13.1	4.48	38.2	15.1	10.6	21.5
(*P* for trend)	<0.001			<0.001			<0.001			<0.001		

Random effects												
Prefectures (*n* = 46)												
Standard deviation	0.283			0.247			1.131			0.426		
(Standard error)	0.074			0.101			0.438			0.113		
Median odds ratio	1.66			1.61			2.76			1.86		

CI, confidence interval; DRCH, designated regional cancer hospital; SHSAs, secondary healthcare service areas.

^a^Designated by Minister of Health, Labour and Welfare in 2012.

## DISCUSSION

This study aimed to describe the distribution of travel time for hospital admission of cancer patients and examine the underlying factors. We observed that patients with cervical cancer or leukemia were generally treated at remote hospitals. Because many medical institutions provide treatment for stomach and colorectal cancers, which are common in Japan, these patients have the option of being admitted to a geographically nearby hospital. However, given the high MORs and marked variations in prefectural characteristics for cervical cancer and leukemia, our results indicate that cancer treatment services for these diseases have been centralized because of the small numbers of patients with these conditions. Consequently, patients with these cancers may have less choice to be treated at closer hospitals compared to patients with other types of cancer.

In terms of equal accessibility to cancer treatment, it is preferable to guarantee geographically easy access to treatment for all cancer patients.^12^ The results of this study suggest that equalizing the accessibility of treatment has been achieved for stomach and colorectal cancers, which are common in Japan, whereas patients with relatively uncommon cancers (eg, cervical cancer and leukemia) tend to be sent to centralized, specialized hospitals. The Ministry of Health, Labour and Welfare has been promoting equalization of cancer medical services.^2^ However, it is necessary to implement a realistic distribution of medical resources depending on cancer type. For example, equalizing the accessibility of treatment for liver and lung cancers (included in the five major cancers) should be discussed with caution because our results show about 23% patients were admitted to hospitals more than a 45-minute drive from their residences. These percentages are higher than that for stomach and colorectal cancers (about 15%). It may be difficult to complete equalization of treatment for liver and lung cancers due to limited medical resources for specialized treatment. Although promoting equalization of cancer medical services is a main purpose of recent health policy in Japan, strategies to balance each patient’s burden and the availability of medical resources are needed.

Sex was not a factor independently impacting travel time exceeding 45 minutes, regardless of cancer sites. On the other hand, the travel time was more likely to exceed 45 minutes in younger patients (Table 3). Thus, the age of cancer patients appears to affect selection of admitting hospitals in terms of geographical access regardless of cancer sites. This raises the possibility that, because younger patients consider various forms of information about medical institutions and tend to select a hospital where they can be actively involved in their own cancer treatment, they are more likely to be treated at geographically remote medical institutions.

This study has some limitations. First, because the expectation values of travel time to a hospital were calculated at the municipality level employing GIS maps with 1-km meshes weighted according to population density, the precise address of each patient was not considered. Second, we applied a single value of 45 minutes as cut-off to define how far hospitals admitting cancer patients are located from their residences. However, we conducted a sensitivity analysis across 30, 45, and 60 minutes for cut-off values, which showed similar results across age-sex groups. The main results, which were shown by multilevel logistic model analysis (Table 3), are also consistent across all cut-off values (30, 45, and 60 minutes); that is, selection of hospitals by cancer inpatients was associated with age, cancer sites, and availability of designated cancer care hospitals regardless of the cut-off used. In addition, approximately 80% of cancer patients in our data were admitted to hospitals less than a 45-minute drive from their residences. The guideline for formulation of medical care plan sets 80% as a threshold for self-containment rate when defining secondary healthcare service area.^13^ When we put together these points, we consider that adopting the single cut-off value was reasonable and that this procedure may not have distorted our results. Third, the present study design was cross-sectional, so a reverse causal relationship can occur. However, it seems unlikely that patients would move to a location near hospitals admitting them only for cancer treatment. Fourth, clinical factors that could have affected selection of admitting hospitals, such as cancer severity and treatment modalities, were not considered.

In conclusion, the majority of cancer patients (approximately 80%) in the database were admitted to hospitals less than a 45-minute drive from their residences. Although age, cancer sites, availability of designated cancer care hospitals in the secondary healthcare service areas where patients live, and population density affect selection of admitting hospitals, our results suggest that sex may not independently affect the selection. When development of a cancer treatment system is planned, geographic aspects must be taken into consideration to guarantee equal access to healthcare for cancer patients.

## APPENDIX A. SUPPLEMENTARY DATA

eTable 1. The percentages of patients estimated travel time exceeding 45 minutes across prefectures (all cancers)
