# Supplementary material for: Geographic Access to Cancer Treatment in Japan: Results From a Combined Dataset of the Patient Survey and the Survey of Medical Institutions in 2011
Source: J Epidemiol. 2018 Nov 5;28(11):470–5. doi: 10.2188/jea.JE20170051 (PMC6192973; doi:10.2188/jea.JE20170051)
Supplement: Supplementary file 1 [file je-28-470-s001.pdf]

eTable 1. The percentages of patients estimated travel time exceeding 45 minutes across prefectures (all cancers)

Region	Prefecture	%	Region	Prefecture	%
Total		22	Kinki		
Hokkaido				Mie	28
	Hokkaido	25		Shiga	21
Tohoku				Kyoto	15
	Aomori	21		Osaka	10
	Iwate	33		Hyogo	24
	Miyagi	25		Nara	21
	Akita	27		Wakayama	22
	Yamagata	15	Chugoku		
	Fukushima	-		Tottori	16
Kanto				Shimane	31
	Ibaraki	34		Okayama	28
	Tochigi	29		Hiroshima	21
	Gumma	23		Yamaguchi	25

	Saitama	27	Shikoku		
	Chiba	30		Tokushima	23
	Tokyo	18		Kagawa	17
	Kanagawa	19		Ehime	31
Chubu				Kochi	38
	Niigata	22	Kyushu		
	Toyama	12		Fukuoka	16
	Ishikawa	26		Saga	24
	Fukui	30		Nagasaki	18
	Yamanashi	26		Kumamoto	27
	Nagano	21		Oita	29
	Gifu	21		Miyazaki	25
	Shizuoka	26		Kagoshima	31
	Aichi	11	Okinawa		
				Okinawa	13
